# *Streptococcus agalactiae* isolated from clinical mastitis cases on large dairy farms in north China: phenotype, genotype of antimicrobial resistance and virulence genes

**DOI:** 10.3389/fcimb.2024.1417299

**Published:** 2024-09-04

**Authors:** Kai Liu, Xiang Liu, Jieyan Yang, Xiaolong Gu, Limei Zhang, Weijie Qu

**Affiliations:** ^1^ College of Veterinary Medicine, Yunnan Agricultural University, Kunming, China; ^2^ Faculty of Animal Science and Technology, Yunnan Agricultural University, Kunming, China

**Keywords:** bovine mastitis, *Streptococcus agalactiae*, antimicrobial resistance, virulence gene, AMR genes detection

## Abstract

*Streptococcus agalactiae* (*Strep. agalactiae*) is bovine mastitis pathogen and has thus became a matter of concern to dairy farms worldwide in terms of economic loss. The aims of this study were to (a) determine virulence genes, and (b) characterize the antimicrobial resistance (AMR) profiles and AMR genes and (c) figure out the relationship between AMR phenotypes and genotypes of *Strep. agalactiae* isolated from dairy cows in north China. A total of 20 virulence genes and 23 AMR genes of 140 isolates collected from 12 farms in six provinces were studied. The antimicrobial susceptibility of 10 veterinary commonly used antimicrobials were tested using the broth microdilution method. Results showed that all the isolates harbored the virulence genes *lac*IV, *gapC*, and *dltA*. The isolates that harbored the genes *lacIII*, *fbsA*, *hylB*, and *cfb* exhibited the high prevalence (99.29%), followed by isolates that harbored *lac*I (98.57%), *bibA* (97.86%), *cylE* (97.14%), *lac*II (92.14%), *cspA* (52.14%), *pavA* (25%), *bca* (2.14%), and *scpB* (0.71%). The *fbsB*, *lmb*, *spbI*, *bac*, and *rib* genes were not detected. The virulence patterns of B (*fbsA*_*cfb*_*cylE*_ *hylB*_*bibA*_*cspA*_ *gapC*_*dltA*_*lacIII/IV*) and C (*fbsA*_*cfb*_ *bibA* _ *gapC*_ *dltA*_*lacIV*) were dominant, accounting for 97.86% of the isolates. The following AMR genes were prevalent: *pbp1A* (97.14%), *tet*(M) (95.00%), *lnu* (A) (80.71%), *erm* (B) (75.00%), *tet*(O) (72.14%), *blaZ* (49.29%), *tet*(S) (29.29%), *blaTEM* (25.71%), *erm* (A) (17.14%), *erm* (C) (13.57%), *tet* (L) (10.71%), *linB* (2.86%), and *erm* (TR) (2.86%). The *pbp2b*, *mecA1*, *mecC*, *lnu* (D), *erm* (F/G/Q), and *mef* (A) genes were not detected. Eighty percent of the isolates harbored AMR genes and were highly resistant to tetracycline, followed by macrolides (10.71%), lincosamides (9.29%) and β-lactams (4.29%). In conclusion, isolates only exhibited well correlation between tetracyclines resistance phenotype and genotype, and almost all isolates harbored intact combination of virulence genes.

## Introduction

Bovine mastitis is one of the costliest diseases in the dairy industry due to the discarding of milk, costs of treatments, and even the culling of cows (Shaheen et al., 2016; Krömker and Leimbach, 2017; Gussmann et al., 2019). *Streptococcus agalactiae* (*Strep. agalactiae*), as one of the major mastitis pathogens, causing 11%–60% of mastitis cases in Brazil (Reyes et al., 2017). The implementation of the five-point mastitis control program has reduced the prevalence of *Strep. agalactiae* mastitis to less than 10% in dairy herds in Europe and North America (Jørgensen et al., 2016). However, the herd prevalence of *Strep. agalactiae* is still high in developing countries, such as Colombia (34.4%), Brazil (60%), and China (92%) (Ramírez et al., 2014; Bi et al., 2016; Carvalho-Castro et al., 2017). Meanwhile, the re-emergence of *Strep. agalactiae* mastitis in Denmark and Norway has been reported (Katholm et al., 2012; Jørgensen et al., 2016). Moreover, the harm caused by Streptococcus agalactiae to China’s dairy farming industry is still very serious (Yang et al., 2016).

*Strep. agalactiae* is considered one of contagious pathogens that cause bovine mastitis, which can spread among cows (Thompson-Crispi et al., 2014). Once *Strep. agalactiae* colonize the bovine mammary gland, it obtains nutrient sources from milk for its proliferation and causes long-term and harmful effects. Therefore, the ability of metabolism and capability for adhesion, invasion, and immune evasion of *Strep. agalactiae* might play crucial roles in the bovine mastitis (Keefe, 2012). *Strep. agalactiae* harbors a great range of virulence genes encoding virulence factors, such as *fbsA/B* and *lmb*, which are involved in adhesion, *cylE* and *hylB*, which are involved in invasion, *cspA*, which is involved in immune evasion, and *LacI/II/III/IV*, which play a role in metabolism.

Antimicrobial treatment is major option for treating *Strep. agalactiae* inducing mastitis (Keefe, 2012). However, the excessive use of antimicrobials increased the risks of antimicrobial resistance (AMR), which is a public health concern worldwide (Flynn and Guarner, 2023). Monitoring the resistance of *Strep. agalactiae* associated with bovine mastitis is important to the control of AMR of the bacterium.

Antimicrobial resistance genes, *pbp1A*, *lnuA*/*D*, *tetO/M/L/S*, *ermA/B/C/F/G/Q/TR*, and *mefA*, which are involved in resistance to β-lactams, lincosamide, tetracycline, and macrolide have been detected in *Strep. agalactiae* usually (Poyart et al., 2003; Dogan et al., 2005; Duarte et al., 2005), while AMR gene carrying status of the strains involved in this study is still unclear.

Investigations on virulence genes, and the phenotype and genotype of AMR can contribute to treatment decision and optimization of *Strep. agalactiae* control programs (Kaczorek et al., 2017). This study aims 1) to determine the antimicrobial resistance and virulence gene profiles of *Strep. agalactiae*, 2) to detect the AMR profiles of *Strep. agalactiae* under *in vitro* conditions, and 3) to determine the correlation between phenotypic and genotypic resistance patterns of *Strep. agalactiae* isolated in China.

## Materials and methods

### Statement of ethics

All experiments followed the China Ministry of Science and Technology. Regulations of Experimental Animals (2008) issued by China Ministry of Science and Technology. All animal procedures were approved by the Institutional Animal Care and Use Committee of Yunnan Agricultural University (Approval No: 202403058).

### Sample collection and identification of pathogens

Milk samples were collected from cows with clinical mastitis from large dairy farms (>500 cows) in China from 2017 to 2019 (**Supplementary Table S1**). Milk sampling details were provided by Gao et al. (2017). In brief, udders were disinfected before sample collection, the first three streams of milk were discarded, and 1–2 mL of quarter milk samples were aseptically collected using 50 mL sterile centrifuge tubes. The samples were packed in ice boxes and delivered to the laboratory to be processed within 10 h.

The quarter milk (200 uL) of each sample was coated on Edwards medium (Oxoid, USA) and incubated at 37°C for 24 h, and putative blue colonies without fermentation were enriched in 4 mL of Mueller–Hinton broth containing 5% fetal bovine serum. The putative isolates were identified through PCR using 16S rRNA amplification with the primer (5’-AGAGTTTGATCCTGGCTCAG-3’, 5’-CGGCTACCTTGTTACGACTT-3’) concentration of 5 μmol/L (Frank et al., 2008). The confirmed *Strep. agalactiae* isolates were stored at −80°C.

### Virulence gene identification

Multiplex PCR was conducted three times for the examination of 13 virulence genes (*csp*A, *pav*A, *cylE*, *hyl*B, *lmb*, *fbsB*, *scp*B, *bca*, *pbp1A/ponA*, *bac*, *cfb*, *rib*, and *fbsA*). The final volume of the multiplex PCR mixture was 25 µL, and the mixture contained the template composed of 1 μL (final amount of 20 ng) of bacterial genome, 12.5 μL of premixed 2×PCR master mix (Sangon, Shanghai, China), 1 μL of each primer (final concentration of 5 μmol/L), and ddH_2_O. The amplification program is provided in **Supplementary Table S1**, in detail, for SET 1 (*csp*A, *pav*A, *cylE*, *hyl*B, *lmb*), the amplification program was as follows: 95°C for 5 min; 35 cycles of 95°C for 60 s; annealing temperature for 60 s; and 72°C for 10 min; for SET 2 (*fbsB*, *scp*B, *bca*), the amplification program was as follows: 95°C for 5 min; 35 cycles of 95°C for 60 s; annealing temperature for 60 s; and 72°C for 10 min; for SET 3 (*pbp1A/ponA*, *bac*, *cfb*, *rib*, and *fbsA*), the amplification program was as follows: 95°C for 5 min; 35 cycles of 95°C for 60 s; annealing temperature for 60 s; and 72°C for 10 min.

The rest of the virulence genes (*spb1*, *dltA*, *bibA*, *gapC*, and *lacI*/*II*/*III*/*IV*) were detected using normal PCR assay. The final volume of the PCR mixture was 25 µL, and the mixture contained the template composed of 1 μL (final amount of 20 ng) of bacterial genome, 12.5 μL of premixed 2×PCR master mix (Sangon, Shanghai, China), 1 μL of primers (final concentration of 5 μmol/L), and 10.5 μL of ddH2O. The amplification program was as follows: 95°C for 5 min; 35 cycles of 95°C for 60 s; annealing temperature for 60 s; and 72°C for 10 min (**Supplementary Table S2**). Strep. agalactiae ATCC 13813 was used as positive control and PCR mixture without bacterial genome was used as negative control. Agarose gel electrophoresis (AGE) and UV transillumination was conducted to analyze the PCR products (**Supplementary Figure S1**). The virulence genes were divided into four groups: adhesion(*fbsA*/*B*, *lmb*, *pavA*), invasion(*cfb*, *cylE*, *hylB*, *spbI*), immune evasion(bac, bca, bibA, cspA, rib, scpB), and metabolism(*gapC*, *dltA*, *LacI*/*II*/*III*/*IV*).

### Antimicrobial resistance gene identification

AMR genes associated with resistance to four kinds of antimicrobials: β_lactams (*blaTEM*, *blaZ, pbp2b*, *mecA1*, and *mecC*), lincosamides (*lnu*A, *lnu*D, and *linB*), tetracyclines (*tet*O/M/L/S), and macrolides (*erm*A/B/C/F/G/Q/TR, *mef*A) were detected using normal PCR assay. The final volume of the PCR mixture was 25 µL containing a template composed of 1 μL (final amount of 20 ng) of bacterial genome, 12.5 μL of premixed 2×PCR master mix (Sangon, Shanghai, China), 1 μL of primers (final concentration of 5 μmol/L), and 10.5 μL of ddH_2_O. The amplification program was as follows: 95°C for 5 min; 35 cycles of 95°C for 60 s; annealing temperature for 60 s; and 72°C for 10 min (**Supplementary Table S3**). *Strep. agalactiae* ATCC 13813 was used as positive control and PCR mixture without bacterial genome was used as negative control. Agarose gel electrophoresis (AGE) and UV transillumination was conducted to analyze the PCR products (**Supplementary Figure S2**).

### Antimicrobial resistance testing

Antimicrobial resistance testing of all the isolates were conducted using the broth microdilution method according to Clinical and Laboratory Standards Institute (CLSI, 2020). *Strep. pneumonia* ATCC 49619 and *Strep. agalactiae* ATCC 13813 were used as quality control strains. Antimicrobials commonly used in practice for mastitis treatment and in medicines for humans (penicillin, cefalexin, ceftiofur, cefquinome, oxacillin, clindamycin, tetracycline, enrofloxacin, amoxicillin/clavulanate, and erythromycin) were selected for antimicrobial resistance testing.

### Statistical analysis

The online statistical tool VassarStats (http://www.vassarstats.net/) was used in calculating the proportion of genes and its 95% confidence interval (95% CI). Correlation calculation was performed using SPSS 26.0 (IBM Corp, Armonk, NY). The cluster of AMR genes and virulence genes were obtained using R (version 4.0.5) and the package “pheatmap” (the clustering method of “complete” and “ward.D” were used).

## Results

### Detection and pattern of virulence genes

The virulence genes were divided into four groups: adhesion, invasion, immune evasion, and metabolism. The dominant virulence genes in the adhesion group were *fbsA* (99.29%; n=139) and *pavA* (25%, n=35), and *cfb*, *cylE*, *gapC*, and *hylB* genes were the predominant invasion genes, accounting for 99.29% (n=139), 97.14% (n=136), 100% (n=140), and 99.29% (n=139), respectively. *bibA*, *cspA*, *bca*, and *scpB* existed in the immune evasion group, with detection rate of which were 97.86% (n=137), 52.14% (n=73), 2.14% (n=3), and 0.71% (n=1), respectively. *dltA* was exhibited in all the isolates (n=140), and detection rate of Lac I/II/III/IV genes were 98.57% (n=138), 92.14% (n=129), 99.29% (139), and 100% (n=140), respectively. *fbsB* and *lmb* were not detected in the adhesion group, *spbI* was not detected in the invasion group, and *bac* and *rib* were not detected in the immune evasion group (**Table 1**).

**Table 1:** Prevalence of virulence genes of 140 *Strep.agalactiae*.

Function	Genes	No. of isolates	Prevalence	95% CI
Adhesion	*fbsA*	139	99.29%	96.07%_99.88%
	*fbsB*	0	0.00%	0%_2.67%
	*lmb*	0	0.00%	0%_2.67%
	*pavA*	35	25.00%	18.56%_32.78%
Total		139	99.29%	96.07%_99.88%
Invasion	*cfb*	139	99.29%	96.07%_99.88%
	*cylE*	136	97.14%	92.88%_98.88%
	*hylB*	139	99.29%	96.07%_99.88%
	*spbI*	0	0.00%	0%_2.67%
Total		140	100.00%	97.33%_100%
Immune evasion	*bac*	0	0.00%	0%_2.67%
	*bca*	3	2.14%	0.73%_6.11%
	*bibA*	137	97.86%	93.89%_99.27%
	*cspA*	73	52.14%	43.92%_60.25%
	*rib*	0	0.00%	0%_2.67%
	*scpB*	1	0.71%	0.12%_3.93%
Total		137	97.86%	93.89%_99.27%
Metabolism	*gapC*	140	100.00%	97.33%_100%
	*dItA*	140	100.00%	97.33%_100%
	*LacI*	138	98.57%	94.94%_99.61%
	*LacII*	129	92.14%	86.48%_95.55%
	*LacIII*	139	99.29%	96.07%_99.88%
	*LacIV*	140	100.00%	97.33%_100%
Total		140	100.00%	97.33%_100%

**Figure 1:**
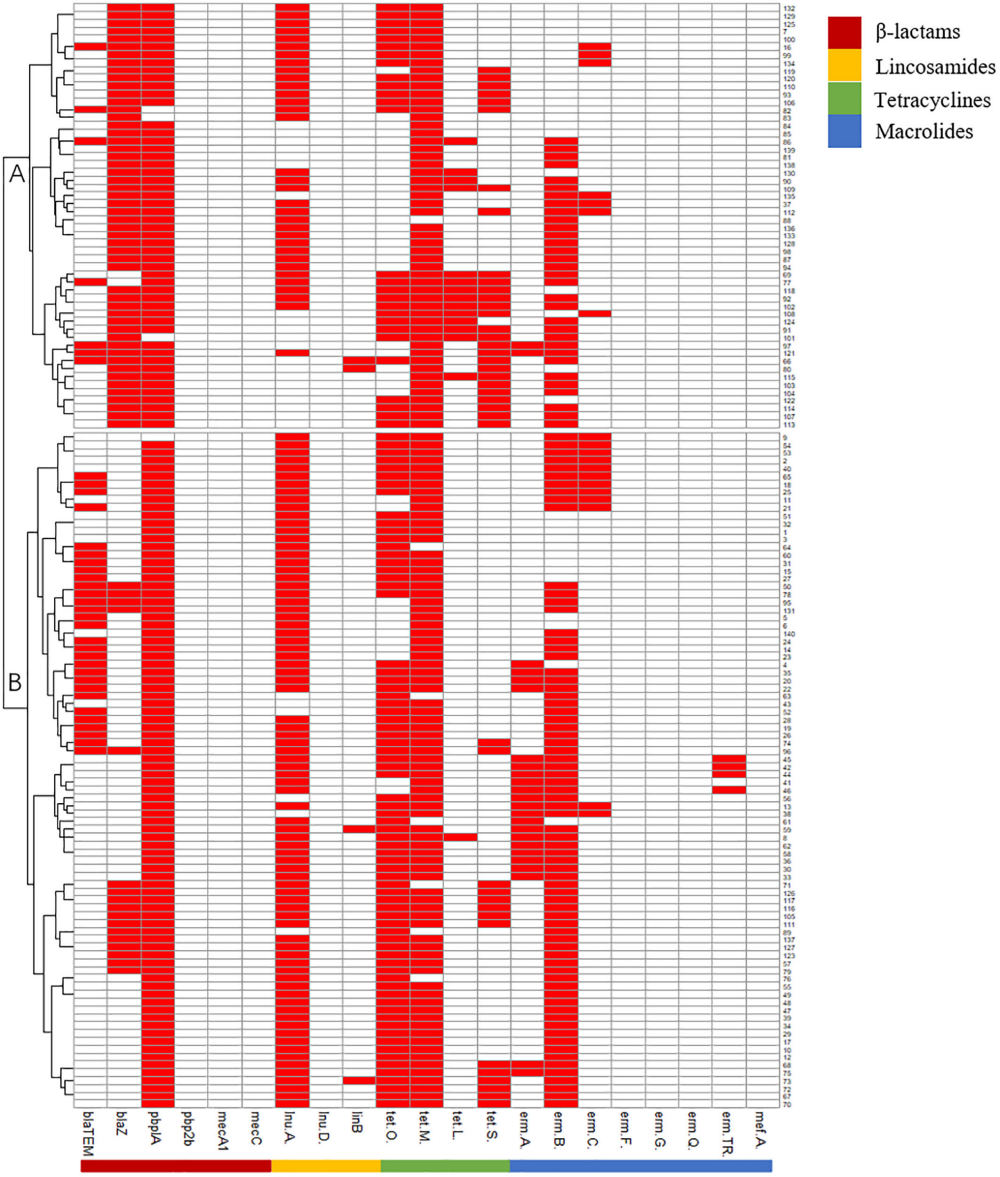
Pattern of virulence genes of 140 *Strep.agalactiae*. white square means absence of virulence genes, red square means presence of virulence genes.

Virulence genes can be grouped into subgroups A (*cylE*_*hylB*_*gapC*_*dltA*_*lacI/II/III/IV*), B (*fbsA*_*cfb*_*cylE*_*hylB*_*bibA*_*cspA*_*gapC*_*dltA*_*lacIII/IV*), and C (*fbsA*_*cfb*_*bibA_gapC*_*dltA*_*lacIV*). Subgroups B and C were the predominant subgroups, accounting for 97.86% of the isolates (**Figure 1**).

### Antimicrobial resistance testing

The isolates were sensitive to most of the tested antimicrobials: penicillin, ceftiofur, Amoxi/clav, and cefquinome (100%); cefalexin (97.9%); oxacillin (96.4%); enrofloxacin (95.7%); erythromycin (89.3%); and clindamycin (88.6%), but only 19.3% of the isolates were sensitive to tetracycline (Liu et al., 2022).

### Detection and patterns of antimicrobial resistance genes

The antimicrobial resistance (AMR) genes were divided into four groups: β-lactam, lincosamide, tetracycline, and macrolide groups according to the type of antimicrobials. The main AMR genes in the β-lactam group were *pbp1A* (97.14%; n=136), *blaZ* (49.29%; n=69), and *blaTEM* (25.71%; n=36). *lnu*(A) and *linB* existed in the lincosamide group, with detection rate of 80.71% (n=113) and 2.86% (n=4), respectively. *tet*(O/M/L/S) were all found in the tetracycline group, with detection rate of 72.14% (n=101), 95.00% (n=133), 10.71% (n=15), and 29.29% (n=41), respectively. In the macrolide group, *erm*(B) was dominant, with a detection rate of 75.00% in the isolates (n=105), followed by *erm*(A), *erm*(C) and *erm*(TR), with detection rate of 17.14% (n=24), 13.57% (n=19), and 2.86% (n=4), respectively. *pbp2b*, *mecA1*, and *mecC* were not detected in the β-lactam group, *lnu*(D) gene was not detected in the lincosamide group, and *erm*(F/G/Q) and *mef*(A) were not detected in the macrolide group (**Table 2**).

**Table 2:** Prevalence of antimicrobials resistant genes of 140 *Strep.agalactiae*.

Antimicrobials	Genes	No. of isolates	Prevalence	95% CI
β_lactams	*blaTEM*	36	25.71%	19.19%_33.53%
	*blaZ*	69	49.29%	41.14%_57.48%
	*pbplA/ponA*	136	97.14%	92.88%_98.88%
	*pbp2b*	0	0.00%	0%_2.67%
	*mecA1*	0	0.00%	0%_2.67%
	*mecC*	0	0.00%	0%_2.67%
Total		139	99.29%	96.07%_99.88%
Lincosamides	*lnu(A)*	113	80.71%	73.39%_86.39%
	*lnu(D)*	0	0.00%	0%_2.67%
	*linB*	4	2.86%	1.12%_7.12%
Total		116	82.86%	75.76%_88.2%
Tetracyclines	*tet(O)*	101	72.14%	64.2%_78.9%
	*tet(M)*	133	95.00%	90.04%_97.56%
	*tet(L)*	15	10.71%	6.6%_16.92%
	*tet(S)*	41	29.29%	22.39%_37.3%
Total		139	99.29%	96.07%_99.88%
Macrolides	*erm(A)*	24	17.14%	11.8%_24.24%
	*erm(B)*	105	75.00%	67.22%_81.44%
	*erm(C)*	19	13.57%	8.86%_20.22%
	*erm(F)*	0	0.00%	0%_2.67%
	*erm(G)*	0	0.00%	0%_2.67%
	*erm(Q)*	0	0.00%	0%_2.67%
	*erm(TR)*	4	2.86%	1.12%_7.12%
	*mef(A)*	0	0.00%	0%_2.67%
Total		120	85.71%	78.96%_90.55%

**Figure 2:**
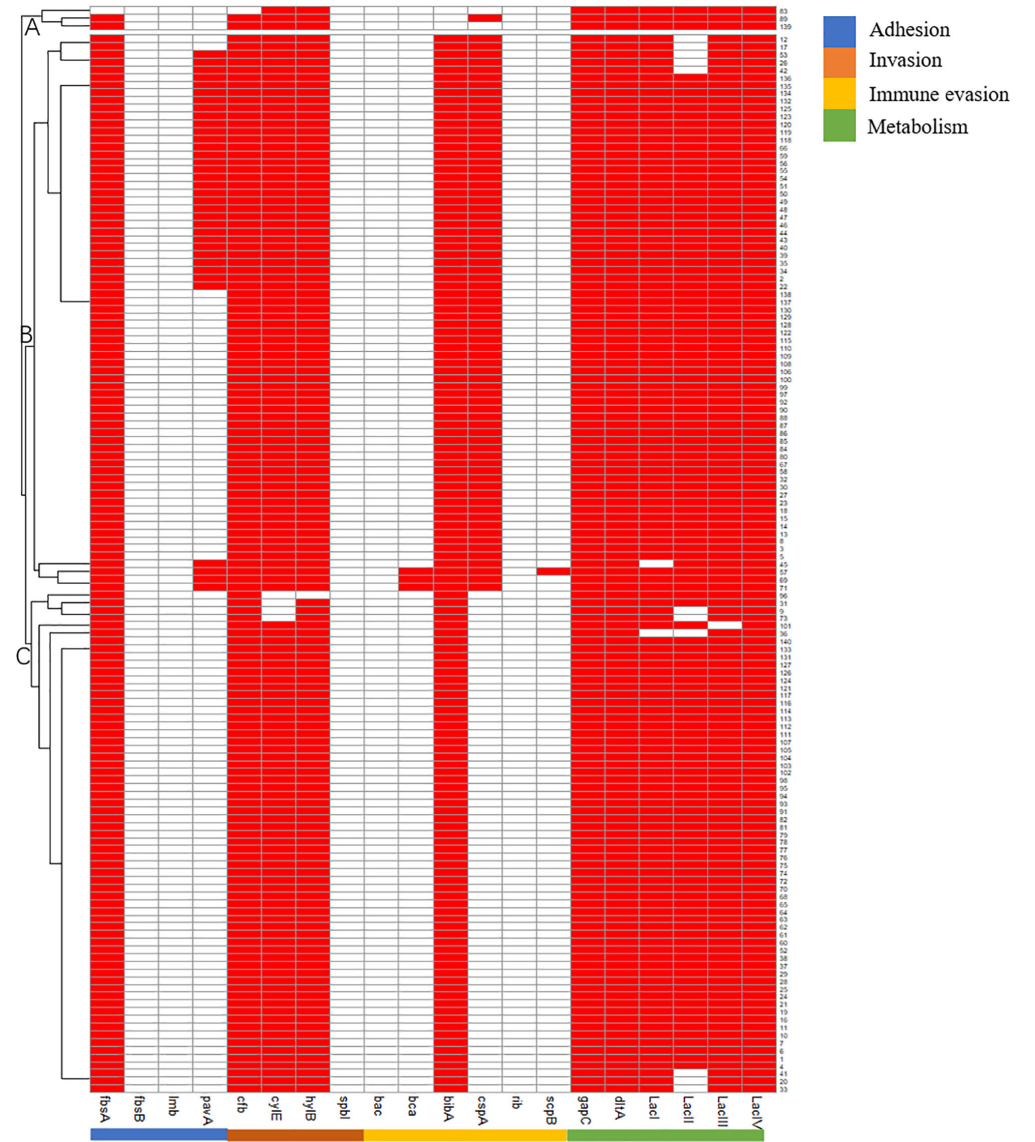
Pattern of antimicrobials resistant genes of 140 *Strep.agalactiae*. Note: white square means absence of antimicrobials resistant genes, red square means presence of antimicrobials resistant genes.

AMR genes can be divided into subgroups A and B. Subgroup A harbored more *blaZ* and *tetL* genes than subgroup B, and subgroup B harbored more *blaTEM* and *ermA*genes than subgroup A (**Figure 2**).

### Relationship between AMR genotype and AMR phenotype

Four types of relationships between the phenotypes and genotypes of the five classes of antimicrobials were examined: resistant phenotype vs presence of ARG (+/+), sensitive phenotype vs presence of ARG (-/+), resistant phenotype vs absence of ARG (+/-), and sensitive phenotype vs absence of ARG (-/-). The pattern in which resistant phenotype vs absence of ARG (+/-) was dominant in the β-lactam, lincosamide, tetracycline, and macrolide groups, accounting for 95% (n=133), 73.57% (n=103), 19.29% (n=27), and 75% (n=105), respectively. In the tetracycline group, 80% of the isolates (n=112) were positive in AMR genes harboring AMR. Only 4.29% (n=6), 9.29% (n=13), and 10.71% (n=15) of the isolates were resistant phenotype and harboring AMR genes in the β-lactam, lincosamide, and macrolide groups. 0.71% (n=1), 15.71% (n=22), 0.71% (n=1), and 14.29% (n=20) of the isolates exhibited sensitive phenotype and absence of ARG in the β-lactam, lincosamide, tetracycline, and macrolide groups. In the lincosamides group, two isolates, accounting for 1.43%, did not harbor any AMR genes but exhibited resistance to clindamycin (**Table 3**).

**Table 3:** Relationship between AMR genotype and AMR phenotype of 140 *Strep.agalactiae*.

Antimicrobials	AMR genotype/AMR phenotype	No.	Prevalence	95% CI	Concordance (κ) ^1^
β-lactams	+/+	6	4.29%	1.98%_9.04%	0.383
	-/+	0	0	0_2.67%	
	+/-	133	95%	90.04%_97.56%	
	-/-	1	0.71%	0.12%_3.93%	
Lincosamides	+/+	13	9.29%	5.51%_15.24%	0.726
	-/+	2	1.43%	0.39%_5.06%	
	+/-	103	73.57%	65.71%_80.17%	
	-/-	22	15.71%	10.61%_22.64%	
Tetracyclines	+/+	112	80%	72.61%_85.78%	0.998
	-/+	0	0	0_2.67%	
	+/-	27	19.29%	13.61%_26.61%	
	-/-	1	0.71%	0.12%_3.93%	
Macrolides	+/+	15	10.71%	6.6%_16.92%	0.684
	-/+	0	0	0_2.67%	
	+/-	105	75%	67.22%_81.44%	
	-/-	20	14.29%	9.45%_21.04%	

Interpretation:κ<0 represents poor agreement; 0 <κ<0.20 represents slight agreement; 0.21<κ<0.40 represents fair agreement; 0.41<κ<0.60 represents moderate agreement; 0.61<κ<0.80 represents substantial agreement; 0.81<κ<1.0 represents almost perfect agreement.

## Discussion

A total of 140 *Strep. agalactiae* isolates were collected from 12 large dairy farms in north China. The percentages of isolates harboring the virulence genes of adhesion(*fbsA*/*B*, *lmb*, *pavA*), invasion(*cfb*, *cylE*, *hylB*, *spbI*), immune evasion(bac, bca, bibA, cspA, rib, scpB), and metabolism(*gapC*, *dltA*, *LacI*/*II*/*III*/*IV*). were 99.29%, 100%, 47.86%, and 100%, respectively. The percentages of the isolates harboring antimicrobial resistance genes of β-lactams, lincosamides, tetracyclines, and macrolides were 99.29%, 82.86%, 99.29%, and 85.71%, respectively. 95%, 73.57%, and 75% of the isolates harbored the antimicrobial resistance genes of β-lactams, lincosamides, but macrolides, and they did not show resistance to the corresponding antimicrobials.

Bovine mastitis induced by *Streptococcus* can be divided into four steps: adhesion and colonization on bovine mammary epithelium cells (bMECs), invasion across or into bMECs, immune evasion, and metabolism (Keefe, 2012). The virulence genes of *Strep. agalactiae* can be categorized into four clusters: adhesion, invasion, immune evasion, and metabolism. In this study, the prevalence of adhesion, invasion, and metabolism clusters were relatively high, and the virulence gene of immune evasion accounted for 47.86% of the isolates. The adhesion genes *fbs*A and *bib*A accounted for 99.29% and 97.86% of the isolates, respectively. The main invasion genes were *gap*C (100%), *hyl*B (99.29%), *cfb* (99.29%), and *cyl*E (97.14%). The metabolism genes were conservative, and their detection rate were relatively high (*dltA*, 100%; *LacI*, 98.57%; *LacII*, 92.14%; *LacIII*, 99.29%; and *LacIV*, 100%). The results were consistent with those in previous research (Keefe, 2012; Morach et al., 2018). The high detection rate of these genes indicated that these genes are essential for the development of bovine mastitis.

The virulence genes *fbsA/B* encode fibrinogen-binding proteins, allowing *Strep. agalactiae* to bind to bMECs and extracellular proteins (Gutekunst and Eikmanns, 2004; Tenenbaum et al., 2005; Pietrocola et al., 2006; Buscetta et al., 2014). In a previous study, *lmb* was found to be associated with the adherence of *Strep. agalactiae*, but it was rarely harbored by bovine mastitis isolates (Duarte et al., 2005; Wu et al., 2016). Our results showed that the major virulence gene in charge of adhesion was *fbsA*, accounting for 99.29% (139/140). The low frequency or absence of *pavA*, *fbsB*, and *lmb* indicated that these genes are not essential to the pathogenesis of bovine mastitis. *cfb* encodes the CAMP factor involved in hemolytic activation (Lasagno et al., 2011). The *cspA* gene encoding serine protease and hemolysin encoded by *cylE* play crucial roles in the virulence of *Strep. agalactiae* (Chou et al., 2019). Hyaluronidase encoded by *hylB* promotes *Strep. agalactiae* invasion in host cells and promotes its host tissue-spreading ability (Oviedo et al., 2013; Coleman et al., 2023). Our study was consistent with the studies of Whist and Osterås (2007) and Pang et al. (2017), who indicated that *cfb*, *cylE*, and *hylB* were the main virulence genes of *Strep. agalactiae* (Whist and Osterås, 2007; Keefe, 2012). The high frequencies of virulence genes associated with invasion indicated that these genes *were* essential to induce clinical bovine mastitis for *Strep. agalactiae* (Keefe, 2012).

Immune evasion enables *Strep. agalactiae* to escape from host immunity killing. The α/β-C protein, as a surface protein, facilitates the invasion of *Strep. agalactiae* in cells and resistance to the clearance of phagocyte; the protein is encoded by *bac* and *bca* (Oviedo et al., 2013; Pulido-Colina et al., 2021). *bac* and *bca* usually appear together (Delannoy et al., 2013). The detection rate of *bac* is low in bovine isolates (Duarte et al., 2005). Our results showed the low detection rate of *bac* and *bca*, indicating they were not essential to bovine mastitis pathogenicity. C5a peptidase cleaving human C5a and BibA known as the C4-binding protein are encoded by *scpB* and *bib*A, respectively. Both proteins hamper the complement system, thereby reducing immune killing (Manne et al., 2020; Cullen et al., 2024). Our result indicated that *bib*A is the main virulence gene involved in the immune evasion of *Strep. agalactiae*. However, Duarte et al. (2005) revealed that 66% of *Strep. agalactiae* isolates from bovine harbor *scpB* (Duarte et al., 2005). Rib encoded by *rib* confers the ability of immune evasion and has been found in most isolates that caused invasive infections (Pulido-Colina et al., 2021). Consistent with our study, previous research indicated that only a small part of *Strep. agalactiae* isolated from bovine (20% and 26%) harbors the *rib* gene (B. Jain et al., 2012).

Rohmer et al. (2011) assumed that bacteria evolved to access specific nutrients that hosts provided and develop pathogenicity (Rohmer et al., 2011). *Lac* encodes lactose operon, and *dItA* encodes D-alanylation of lipoteichoic acid, which is involved in the completion of the cell wall of Gram-positive bacteria. The genes were conserved in all *Strep. agalactiae* isolates. Glyceraldehyde-3-phosphate dehydrogenase encoded by *gapC* is involved in carbohydrate metabolism. Our results indicated the ability of *Strep. agalactiae* to use milk as a nutrient resource due to these metabolism genes (Keefe, 2012). Overall, the results of our research indicated that the integrity of the parts of the virulence genes (adhesion, invasion, immune evasion, and metabolism) mediates the pathogenesis of *Strep. agalactiae*.

Isolates were sensitive to most of the tested antimicrobials: penicillin, ceftiofur, amoxi/clav, cefquinome, and vancomycin (100%); cefalexin (97.9%); oxacillin (96.4%); enrofloxacin (95.7%); erythromycin (89.3%); and clindamycin (88.6%). However, only 19.3% of the isolates were sensitive to tetracycline, and 0.7% were sensitive to daptomycin (Liu et al., 2022).

The percentage of the isolates resistant to tetracycline was 80%, in line with the results of Gao et al. (2012) and Tomazi et al. (2018), who reported that the percentages of resistance were 72.5% in China and 68.6% in Brazil, respectively (Gao et al., 2012; Tomazi et al., 2018). The low efficacy of tetracycline in treating mastitis has been reported worldwide, and one of the reasons is its excessive use in treatment and growth promotion (Kaczorek et al., 2017). This antimicrobial should be used prudently in the treatment of mastitis.

In addition to the AMR profiles of *Strep. agalactiae* under *in vitro* conditions, genotypic AMR detection was performed for the selected AMR genes encoding different resistance mechanisms.

The results of our research were consistent with those of Kannika et al. (2017) (Kannika et al., 2017). *pbp*l*A/ponA* (penicillin-binding protein 1A) was the dominant gene encoding resistance to β-lactams and accounted for 97.14%, followed by *blaZ* (49.29%).

The lincosamides resistant genes we detected are the *lnu A/D* and *linB* genes, nucleotidyl transferases are encoded by *lnu* genes, resulting in enzymatic inactivation of lincosamides. The *lnu* gene was first identified in *Enterococcus faecium* and then observed in *Strep. agalactiae* (Arana et al., 2014; Kaczorek et al., 2017). Our results indicated that the detection rate of *lnu*(A) was 80.71%, which may raise concerns about the spreading of AMR genes among bacteria.

We detected four genes responsible for resistance to tetracyclines: *tet*(M), *tet*(O), *tet*(S). and *tet*(L), which encodes resistance through ribosomal protection and efflux pump (Poyart et al., 2003; Dogan et al., 2005; Gao et al., 2012). In our research, the *tet*(M) and *tet*(O) genes were predominant, which is consistent with previous study (Gao et al., 2012; Rato et al., 2013). The high detection rate of these genes can be attributed to horizontal gene transfer in the same genus of bacteria (Gao et al., 2012; Ruegg et al., 2015).

Eight genes encoding macrolide resistance were detected. *erm*(B) was predominant, consistent with previous reports (Loch et al., 2005; Gao et al., 2012; Rato et al., 2013). *erm*(B) can encode methylase, reducing the number of macrolides binding to *Strep. agalactiae* (Denamiel et al., 2005). *erm*(B) can transfer among bacteria in the same genus (Loch et al., 2005), and this feature explains the high detection rate of the gene. *mef*(A), harbored by only 2.86% of isolates, was examined as well. The results were consistent with those of previous research (Rato et al., 2013).

There were two isolates that exhibited resistance to lincosamides but did not harbor the examined AMR genes, the possible reason for which is that we failed to detect other resistance genes that encoding lincosamides resistance. Nevertheless, some isolates exhibited AMR genes carrying but negative in AMR phenotype. The reasons were as follows: (1) AMR genes may not transcribe nor translate because the corresponding antimicrobials were not used in bovine mastitis treatment, so they were far from a promoter or associated with a weak promoter; (2) mutations or lack of promoters induce the silencing of the AMR genes of the isolates. Advanced research is essential to discover the mechanisms of the insufficient correlation between the genotype and phenotype of AMR (Gao et al., 2012).

For the reason of insufficient controls (positive controls for each AMR or virulence genes) were used in our research, the genes that did not be detected may due to the following situations: (1) the isolates did not harbor corresponding genes, (2) the primers failed to combine to template on account of gene mutation.

## Conclusion

Some isolates resistant to lincosamides did not necessarily carry any tested gene. Conversely, a large part of β-lactams, lincosamides, and macrolides sensitive isolates contained corresponding AMR genes, which may not be expressed in these isolates. Furthermore, based on almost all isolates harbored virulence genes encoded the ability of adhesion, invasion, immune evasion and metabolism, we inferred that intact combination of virulence genes is essential to the pathogenesis of *Strep. agalactiae* inducing bovine mastitis.

## Data Availability

The datasets presented in this study can be found in online repositories. The names of the repository/repositories and accession number(s) can be found in the article/**Supplementary Material**.
