# Supplementary material for: Streptococcus agalactiae isolated from clinical mastitis cases on large dairy farms in north China: phenotype, genotype of antimicrobial resistance and virulence genes
Source: Front Cell Infect Microbiol. 2024 Sep 4;14:1417299. doi: 10.3389/fcimb.2024.1417299 (PMC11409094; doi:10.3389/fcimb.2024.1417299)
Supplement: Supplementary file 1 [file Table1.docx]

**STable1. Distribution and prevalence of 140 *Streptococcus aglactiae* isolated from clinical mastitis in 201 large Chinese dairy herds.**

| Province | Herds | | Samples | | *Streptococcus*  spp. | | *Streptococcus aglactiae* | |
| --- | --- | --- | --- | --- | --- | --- | --- | --- |
|  | No. | % | No.^1^ | % | Isol | % | Isol | % |
| Anhui | 6 | 3.0 | 159 | 3.9 | 37 | 23.3 | 0 | 0.0 |
| Beijing | 12 | 6.0 | 243 | 6.0 | 17 | 7.0 | 0 | 0.0 |
| Fujian | 1 | 0.5 | 12 | 0.3 | 0 | 0.0 | 0 | 0.0 |
| Gansu | 1 | 0.5 | 30 | 0.7 | 0 | 0.0 | 0 | 0.0 |
| Guangdong | 2 | 1.0 | 98 | 2.4 | 12 | 12.2 | 0 | 0.0 |
| Hebei | 61 | 30.3 | 867 | 21.5 | 129 | 14.9 | 26 | 3 |
| Henan | 6 | 3.0 | 86 | 2.1 | 6 | 7.0 | 1 | 1.2 |
| Heilongjiang | 18 | 9.0 | 434 | 10.7 | 45 | 10.4 | 7 | 1.6 |
| Hubei | 1 | 0.5 | 55 | 1.4 | 2 | 3.6 | 0 | 0.0 |
| Hunan | 1 | 0.5 | 21 | 0.5 | 3 | 14.3 | 0 | 0.0 |
| Jilin | 1 | 0.5 | 8 | 0.2 | 2 | 25.0 | 0 | 0.0 |
| Jiangsu | 1 | 0.5 | 9 | 0.2 | 3 | 33.3 | 0 | 0.0 |
| Liaoning | 10 | 5.0 | 156 | 3.9 | 19 | 12.2 | 0 | 0.0 |
| Inner Mongolia | 47 | 23.4 | 1334 | 33.0 | 232 | 17.4 | 19 | 1.4 |
| Shandong | 8 | 4.0 | 426 | 3.1 | 93 | 21.8 | 83 | 19.5 |
| Shanxi | 2 | 1.0 | 26 | 0.6 | 1 | 3.8 | 0 | 0.0 |
| Shaanxi | 6 | 3.0 | 68 | 1.7 | 3 | 4.4 | 0 | 0.0 |
| Shanghai | 5 | 2.5 | 108 | 2.7 | 24 | 22.2 | 0 | 0.0 |
| Tianjin | 7 | 3.5 | 92 | 2.3 | 21 | 22.8 | 4 | 4.3 |
| Yunnan | 5 | 2.5 | 109 | 2.7 | 36 | 33.0 | 0 | 0.0 |
| Total | 201 | 100 | 4341 | 100 | 685 | 15.8 | 140 | 3.2 |

^1^Number of clinical mastitis isolates

**STable2. Primers and annealing temperatures of virulence genes.**

| Gene | Primer | Size (bp) | Sequence 5’-3’ | Annealing temperature (°C) | References |
| --- | --- | --- | --- | --- | --- |
| Set 1 | *cspA*-F | 971 | CTGCTAAAGCACACCTAAAC | 47 | Pisuttharachai et al., 2017 |
|  | *cspA*-R |  | ATCAGTAGTGGTTCCTTTCC |  |  |
|  | *pavA*-F | 729 | TACTACCAAGAGAAGGCTGA |  |  |
|  | *pavA*-R |  | GGAGAGACGAGCTTTAGAGT |  |  |
|  | *cylE-*F | 564 | GTACATTAGGTGCCTTTGG |  |  |
|  | *cylE*-R |  | TACTCAGCCTTTCTCCATC |  |  |
|  | *hylB*-F | 323 | TCTATGCTGACGGTTCTTAC |  |  |
|  | *hylB*-R |  | GAGGTCTAAGTTTCGCTCTT |  |  |
|  | *lmb*-F | 152 | TCAGTTAGTTGCTCTGCTTC |  |  |
|  | *lmb*-R |  | CTTTATGACCCACATACCTG |  |  |
| Set 2 | *fbsB*-F | 936 | CACTCGATAACACTGTGGAT | 45 | Pisuttharachai et al., 2017 |
|  | *fbsB*-R |  | CTGGAACTGTTTCTGTCTTG |  |  |
|  | *scpB*-F | 255 | ACAACGGAAGGCGCTACTGTTC |  | Dmitriev *et al.* 2004 |
|  | *scpB*-R |  | ACCTGGTGTTTGACCTGAACTA |  |  |
|  | *bca*-F | 535 | TAACAGTTATGATACTTCACAGAC |  | Manning *et al*. 2006 |
|  | *bca*-R |  | ACGACTTTCTTCCGTCCACTTAGG |  |  |
| Set 3 | *pbp1A/ponA*-F | 939 | AGGGGTAGTAGCATTACCAT | 47 | Pisuttharachai et al., 2017 |
|  | *pbp1A/ponA*-R |  | CAACTATATGACTGGGATCG |  |  |
|  | *bac*-F | 750 | CTCCAAGCTCTCACTCATAG |  |  |
|  | *bac*-R |  | GAAACATCTGCCACTGATAC |  |  |
|  | *cfb*-F | 600 | GGATTCAACTGAACTCCAAC |  |  |
|  | *cfb*-R |  | GACAACTCCACAAGTGGTAA |  |  |
|  | *rib*-F | 425 | GGGGTTACACAAGGTAATCT |  |  |
|  | *rib-R* |  | TCCACTTAGGATCGTTTG |  |  |
|  | *fbsA*-F | 278 | AACCGCAGCGACTTGTTA |  |  |
|  | *fbsA-R* |  | AAACAAGAGCCAAGTAGGTC |  |  |
| *spb1* | *spb1*-F | 648 | CTGCTCCAAGCATAATGCTT | 48 | Pisuttharachai et al., 2017 |
|  | *spb1*-R |  | ACCCATCAGAACCAAAAGT |  |  |
| *dltA* | *dltA_F* | 100 | GTTTTTGGTAGGGCAAACAGGGTGC | 50 | Pereira et al., 2010 |
|  | *dltA_R* |  | CGCAAATGTTGGCTCAACCGCC |  |  |
| *bibA* | *bibA_F* | 127 | AACCAGAAGCCAAGCCAGCAACC | 50 |  |
|  | *bibA_R* |  | AGTGGACTTGCGGCTTCACCC |  |  |
| *gapC* | *gapC_F* | 100 | AGACCGATAGCTTTTGCAGCACC | 50 |  |
|  | *gapC_R* |  | GATCCTTGACGGACCACACCG |  |  |
| Lactose operon | *lacI_F* | 756 | TAATGCTTTCGCAGTCGT | 50 | Pang et al., 2017 |
|  | *lacI_R* |  | GTGCTACTTGGGCAGGAT |  |  |
|  | *lacII_F* | 702 | ACAAATCGCACAAAGAGC | 52 |  |
|  | *lacII_R* |  | CAACTACAGTATCAACACGAGAAT |  |  |
|  | *lacIII_F* | 1374 | ATTGGTGGTGAGTGTCGT | 50 |  |
|  | *lacIII_R* |  | GGTGGCTTCTTGGTATTG |  |  |
|  | *lacIV_F* | 900 | AAGGCTAAGGCAGAAATA | 48 |  |
|  | *lacIV_R* |  | TGGTAAAGGCTTGAATGT |  |  |

**STable3. Primers and annealing temperatures of antimicrobials resistant genes.**

| Gene function | Primer | Size (bp) | Sequence (5′–3′) | Annealing temperature (°C) | Reference |
| --- | --- | --- | --- | --- | --- |
| β-lactams  Ampicillin | *blaZ*_F | 517 | AAGAGATTTGCCTATGCTTC | 45 | Vesterholm-Nielsen et al., 1999 |
|  | *blaZ*_R |  | GCTTGACCACTTTTATCAGC |  |  |
|  | *pbp2b*_F | 1500 | GATCCTCTAAATGATTCTCAGGTGG | 55 | Ding, 2015 |
|  | *pbp2b*_R |  | CCATTAGCTTAGCAATAGGTGTTGG |  |  |
|  | *mecA1*_F | 533 | AAAATCGATGGTAAAGGTTGGC | 54 | Frey Y et al.,2013 |
|  | *mecA1*_R |  | AGTTCTGCAGTACCGGATTTGC |  |  |
|  | *mecC*_F | 486 | AGCCAGATTCATTTGTACC | 54 |  |
|  | *mecC*_R |  | AACATCGTACGATGGGGTAC |  |  |
|  | *blaTEM*_F | 800 | CCGTGTCGCCCTTATTCC | 55 | Raphael E et al.,2011 |
|  | *blaTEM*_R |  | AGGCACCTATCTCAGCGA |  |  |
| quinolones | *gyrA*_F | 496 | CGATGTCGGTCATTGTTG | 50.5 | Schmitt-Van de et al. 2007 |
|  | *gyrA*_R |  | ACTTCCGTCAGGTTGTGC |  |  |
|  | *parC*_F | 567 | CTGAATGCCAGCGCCAAAT | 56 |  |
|  | *parC*_R |  | GCGCATACGCACTGAACC |  |  |
| Lincosamides | *lnu*(D)_F | 475 | ACGGAGGGATCACATGGTAA | 50 | Haenni et al., 2011 |
|  | *lnu*(D)_R |  | TCTCTCGCATAATAACCTTACGTC |  |  |
|  | *lnu*(A)_F | 323 | GGTGGCTGGGGGGTAGATGTATTAACTGG | 56 | Lina et al., 1999 |
|  | *lnu*(A)_R |  | GCTCTCTTTGAAATACATGGTATTTTTCGATC |  |  |
|  | *linB*_F | 925 | CCTACCTATTGTTTGTGGAA | 54 | Desjardins M et al., 2004 |
|  | *linB*_R |  | ATAACGTTACTCTCCTATTC |  |  |
| Tetracyclines | *tet*(O)_F | 515 | AACTTAGGCATTCTGGCTCAC | 50 | Ng et al., 2001 |
|  | *tet*(O)_R |  | TCCCACTGTTCCATATCGTCA |  |  |
|  | *tet*(L)_F | 267 | TCGTTAGCGTGCTGTCATTC | 50 |  |
|  | *tet(*L)_R |  | GTATCCCACCAATGTAGCCG |  |  |
|  | *tet*(M)_F | 406 | GTGGACAAAGGTACAACGAG | 50 |  |
|  | *tet*(M)_R |  | CGGTAAAGTTCGTCACACAC |  |  |
|  | *tet*(S)_F | 667 | CATAGACAAGCCGTTGACC | 48 |  |
|  | *tet*(S)_R |  | ATGTTTTTGGAACGCCAGAG |  |  |
| Macrolides | *erm*(A)_F | 421 | GTTCAAGAACAATCAATACAGAG | 48 | Lina et al., 1999 |
|  | *erm*(A)_R |  | GGATCAGGAAAAGGACATTTTAC |  |  |
|  | *erm*(B)_F | 652 | CGAGTGAAAAAGTACTCAACC | 48 | Villaseñor-Sierra et al., 2012 |
|  | *erm*(B)_R |  | AGTAACGGTACTTAAATTGTTTAC |  |  |
|  | *erm*(C)_F | 295 | ATCTTTGAAATCGGCTCAGG | 47 | Jensen et al., 1999 |
|  | *erm*(C)_R |  | CAAACCCGTATTCCACGATT |  |  |
|  | *erm*(F)_F | 424 | TCT GGG AGG TTC CATTGT CC | 65 | Koike S et al., 2010 |
|  | *erm*(F)_R |  | TTC AGG GAC AAC TTCCAG C |  |  |
|  | *erm*(G)_F | 255 | GTG AGG TAA CTC GTAATA AGCTG | 63 |  |
|  | *erm*(G)_R |  | CCT CTG CCA TTA ACAGCA ATG |  |  |
|  | *erm*(Q)_F | 154 | CAC CAA CTG ATA TGTGGC TAG | 68 |  |
|  | *erm*(Q)_R |  | CTA GGC ATG GGA TGGAAG TC |  |  |
|  | *mef*(A)_F | 500 | AGTATCATTAATCACTAGTGC | 45 | Villaseñor-Sierra et al., 2012 |
|  | *mef*(A)_R |  | TTCTTCTGGTACTAAAAGTGG |  |  |
|  | *erm*(TR)_F | 376 | ATAGAAATTGGGTCAGGAAAAGG | 48 |  |
|  | *erm*(TR)_R |  | CCCTGTTTACCCATTTATAAACG |  |  |


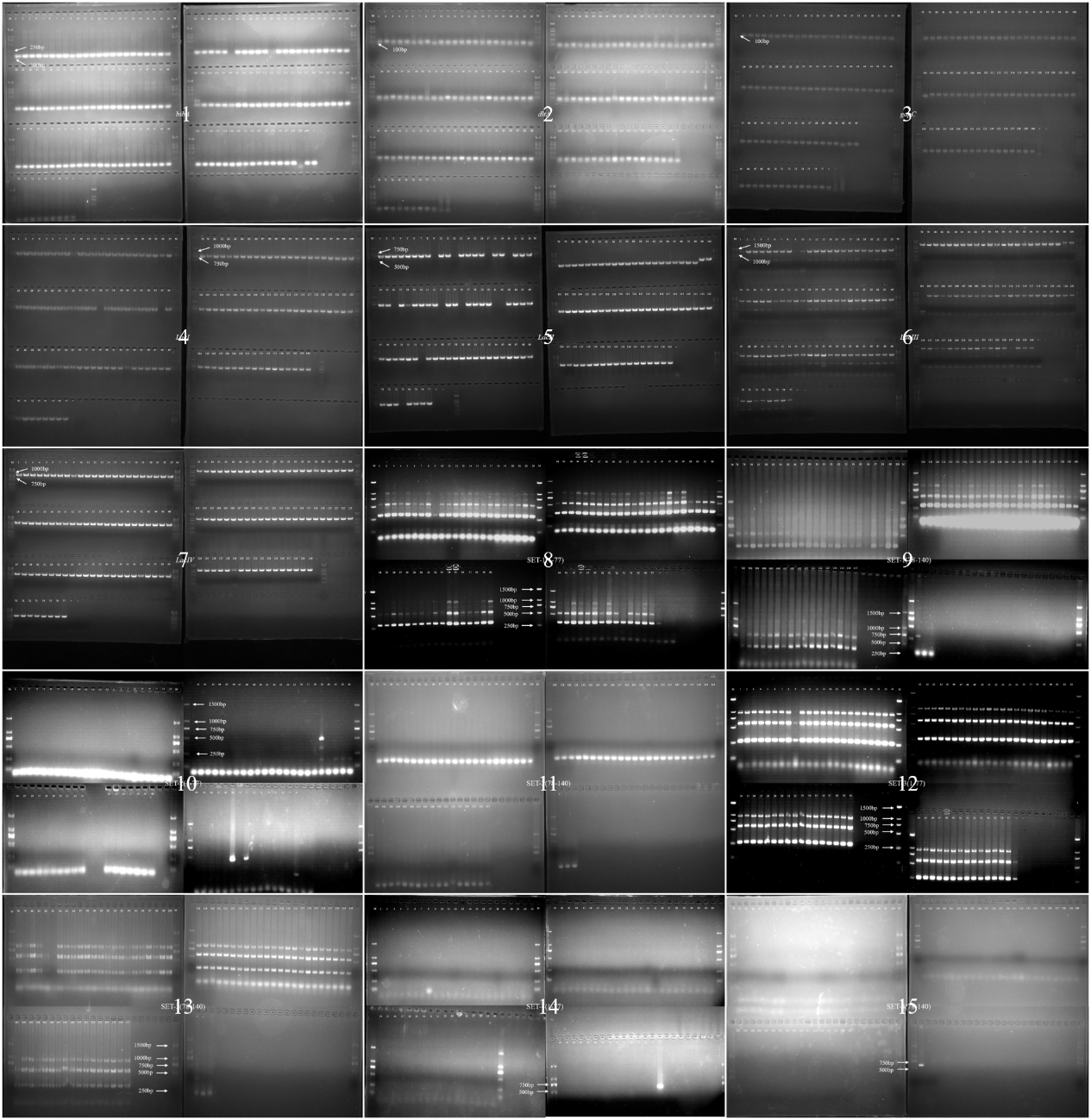


**SFig1. Gel images of virulence genes of *Strep.agalactiae.***

White arrows: the aimed band area; M: DNA marker; 1: *bibA*, 2: *dltA*, 3: *gapC*, 4: *LacI*, 5: *LacII*, 6: *LacIII*, 7: *LacIV*, 8 and 9: SET1, 10 and 11: SET2, 12 and 13: SET3, 14 and 15: SET 4.


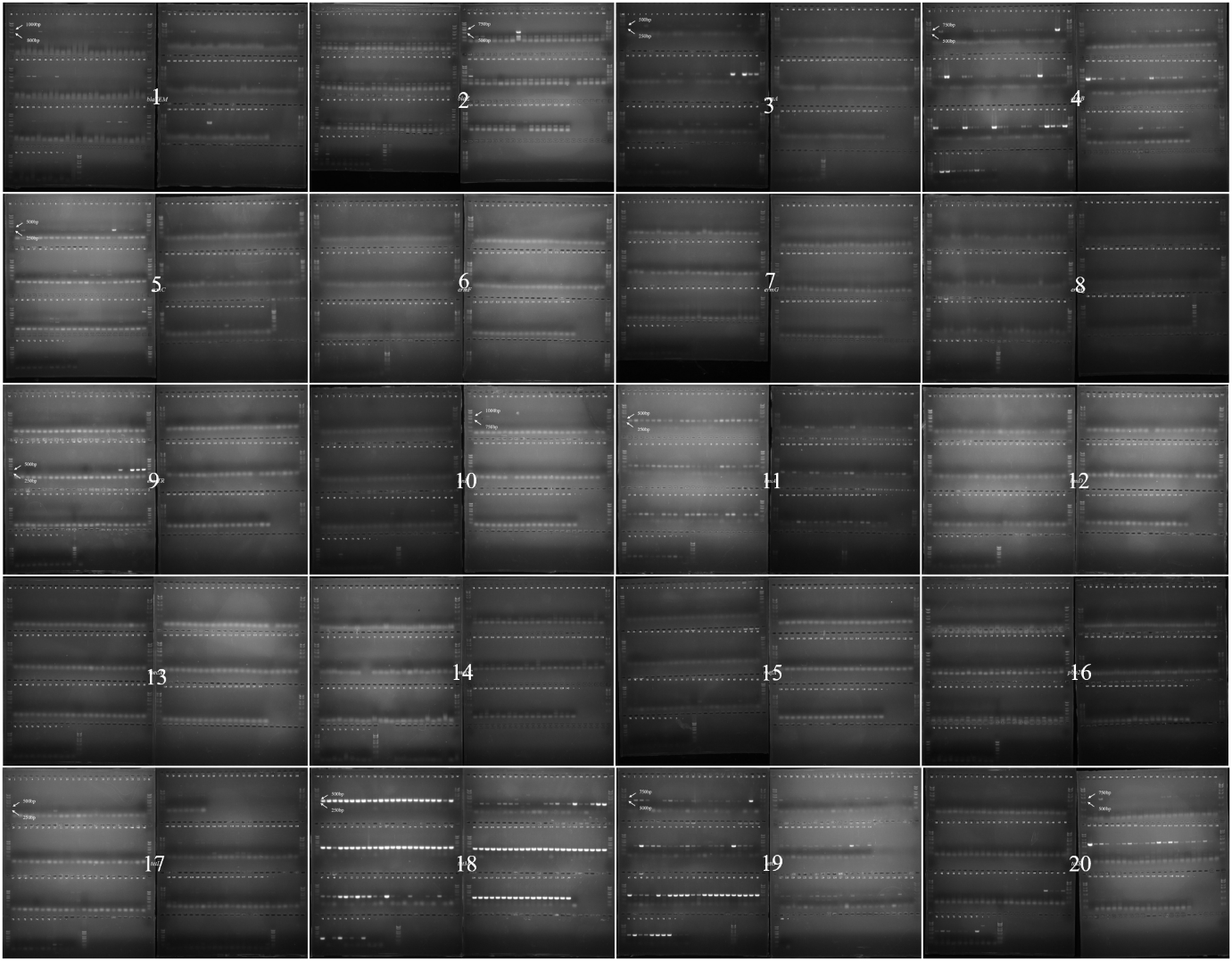


**SFig2. Gel images of antimicrobials resistant genes of *Strep.agalactiae.***

White arrows: the aimed band area; M: DNA marker; 1: *blaTEM*, 2: *blaZ*, 3: *ermA*, 4: *ermB*, 5: *ermC*, 6: *ermF*, 7: *ermG*, 8: *ermQ*, 9: *ermTR*, 10: *linB*, 11: *lnuA*, 12: *lnuD*, 13: *mecA1*, 14: *mecC*, 15: *mefA*, 16: *pbp2b*, 17: *tetL*, 18: *tetM*, 19: *tetO*, 20: *tetS*.
